# Specular Microscopic Features of Corneal Endothelial Vacuolation

**Published:** 2011-01

**Authors:** Mozhgan Rezaei Kanavi, Mohammad-Ali Javadi, Tahereh Chamani

**Affiliations:** 1 Ophthalmic Research Center, Shahid Beheshti University of Medical Sciences, Tehran, Iran; 2 Central Eye Bank of the Islamic Republic of Iran

**Keywords:** Specular Microscopy, Cornea, Endothelium, Vacuole

## Abstract

**Purpose:**

To introduce a specular microscopic reference image for endothelial vacuolation in donated corneas.

**Methods:**

Two corneas from a donor with diffuse, round to oval dark areas at the endothelial level on slit lamp biomicroscopy and one normal-appearing donor cornea underwent specular microscopy, histopathologic evaluation and transmission electron microscopy.

**Results:**

Specular microscopy of the two corneas with abnormal-looking endothelium revealed large numbers of dark, round to oval structures within the endothelium in favor of endothelial vacuolation. Light microscopy disclosed variable sized cyst-like structures within the cytoplasm. Transmission electron microscopy showed electron-lucent and relatively large-sized intracytoplasmic vacuoles. These features were not observed in the endothelium of the normal cornea.

**Conclusion:**

The specular microscopic features of endothelial vacuolation in donated corneas were confirmed by light microscopy and transmission electron microscopy, therefore the specular image may be proposed as a reference to eye banks.

## INTRODUCTION

The integrity of corneal endothelial cells is essential for maintaining their physiologic functions and consequently corneal clarity.^1^ Rapid post-mortem degenerative changes in the corneal endothelium have been reported as progressive development of numerous large vacuoles.^2^ In a donated cornea, preserved under hypothermic conditions in a preservative media, a combination of the effects of cold storage^3^ and post-mortem changes^2^ in the corneal endothelium with consequent loss of endothelial pump function may contribute to formation of dark blister-like structures within the cytoplasm.

Endothelial vacuole formation is one of the indices for corneal rating (EBAA Medical Advisory Board, EBAA Medical Standards, November 1999); the greater the endothelial vacuolation, the lower the corneal quality. The appearance of endothelial cell vacuolation on specular microscopy of donated corneas is a frequently asked question by eye bank technicians, therefore there is a need for a typical reference specular microscopic image for endothelial vacuolation. On the other hand, dark areas seen at the endothelial level may suggest the presence of pigment and/or red blood cells released before or during corneoscleral excision. There are a series of studies on scanning and electron microscopic features of endothelial cell vacuolation in rabbit and cat corneas.^4^–^6^ Herein, we present the specular microscopic features of endothelial vacuolation in donated corneas confirmed by histopathologic evaluation and transmission electron microscopy.

## METHODS

Two corneas from a young male donor with diffuse round to oval dark areas at the endothelial level on slit lamp biomicroscopy underwent specular microscopy, histopathologic evaluation and transmission electron microscopy. The dark areas had focal red- to brown-colored reflections on slit lamp biomicroscopy, suspicious of pigment or red blood cells precipitates. We simultaneously performed specular, light and electron microscopic examinations in a cornea from another young male donor who had normal-appearing endothelium on slit lamp biomicroscopy. Both donors had passed away due to multiple trauma. Death to preservation time had been less than 12 hours in the normal cornea and 31 hours in the corneas with abnormal endothelium. The donated corneas were preserved in Optisol maintenance media (Optisol-GS, Bausch & Lomb Inc., Rochester, NY, USA) at 4°^C^ and then warmed up to room temperature before slit lamp biomicroscopic examination.

After performing specular microscopy (Konan Eye Bank Keratoanalyser, Hyogo, Japan), the corneas were fixed in 2.5% glutaraldehyde and processed for transmission electron microscopy. Semi-thin sections stained with toluidine blue were examined with light microscopy (Olympus BX41, Tokyo, Japan) by an ophthalmic pathologist (MRK). Ultrathin sections stained with uranyl acetate 1% were evaluated with transmission electron microscopy (EM 900, Zeiss, Germany) by the same examiner.

## RESULTS

**Figure 1:**
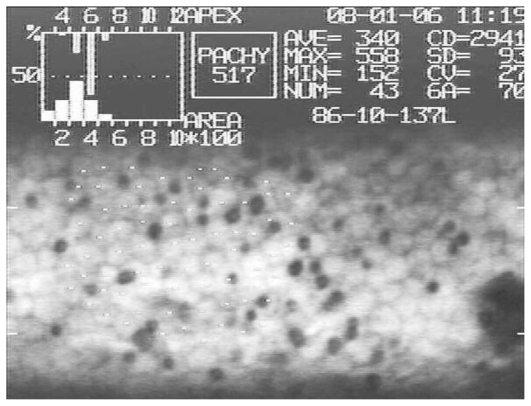
Round to oval dark areas within and between endothelial cells on specular microscopy of the donated cornea with abnormal endothelium.

**Figure 2:**
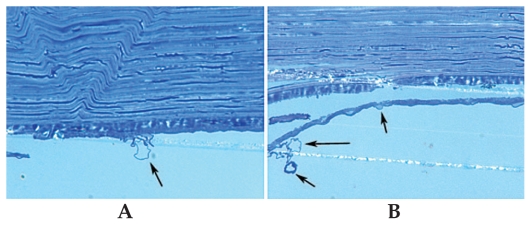
Bleb-like structures (arrows, A & B) within the endothelial cytoplasm near the posterior cytoplasmic membrane, some extruding from the endothelial cell surface (toluidine blue stain, magnification ×1,000).

Specular microscopy of corneas with abnormal endothelium disclosed scattered round to oval non-reflective structures within the endothelium (Fig. 1). Examination of semi-thin preparations of the specimens revealed cyst-like structures measuring 2 to 17 (average 8.6±5.2) μm in maximum diameter within the endothelial cytoplasm, mainly near the posterior cytoplasmic membrane. In some areas the cystic structures coalesced and were extruded from the cell surface (Fig. 2).

**Figure 3:**
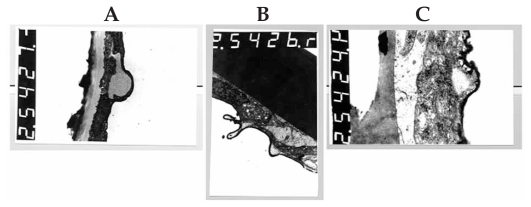
Electron-lucent intracytoplasmic vacuoles protruding from the posterior cytoplasmic membrane of an endothelial cell on transmission electron microscopy of the donated cornea [magnification ×3,000 (A), ×4,400 (B), and ×12,000 (C)].

**Figure 4:**
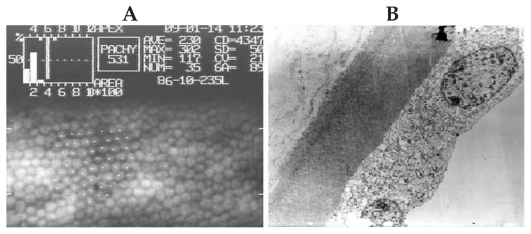
Note absence of dark areas on specular microscopy **(A)** and transmission electron microscopy **(B)** of the cornea with normal endothelial cells (magnification ×3,000).

Transmission electron microscopy confirmed the specular microscopic findings by disclosing foci of protruded posterior cytoplasmic membrane in the endothelial cells due to presence of electron-lucent intra-cytoplasmic vacuoles (Fig. 3). These micro- and ultra-structural features were not observed in the cornea with normal-appearing endothelium (Fig. 4) which was rated excellent based on slit lamp and specular microscopic findings.

## DISCUSSION

Through histopathologic and electron microscopic evaluations, we were able to confirm that the dark round to oval areas observed on specular microscopy of the endothelium in donated corneas actually represented endothelial vacuolation. Such findings were not observed on transmission electron microscopy of a control cornea with normal endothelium. On slit lamp biomicroscopy of the affected corneas, the dark endothelial areas had a red- to brown- reflection raising the suspicion of pigment or red blood cell precipitates. Given the results of light and transmission electron microscopy, such hue in the endothelial vacuoles may be due to reflection of the phenol red indicator present in Optisol from the surface of the vacuoles.

The occurrence of dark blister-like structures in the cytoplasm of endothelial cells may be attributed to the reversible effect of cold storage^3^ but their persistence after warming up may suggest irreversible post-mortem bleb formation.^2^ Postmortem endothelial cell bleb formation is a component of apoptosis and is accompanied by characteristic cellular and molecular degenerative changes.^7^,^8^ It seems that death to preservation time may contribute to the evolution of postmortem changes including vacuole formation; the less this time, the less endothelial vacuolization. In the current report donated corneas with significant endothelial vacuole formation had longer death to preservation time than the normal cornea.

Vacuole formation in the endothelium is one of the criteria for corneal quality rating in eye banking and the presence of marked endothelial vacuolation leads to lower corneal quality rating (EBAA Medical Advisory Board, EBAA Medical Standards, November 1999). For eye bank technicians, a typical specular microscopic reference image depicting features of endothelial vacuolation is necessary for such rating; this study may provide such a reference image to other eye banks.

In summary, dark endothelial areas detected on specular microscopy of donated corneas were confirmed to be vacuoles based on light microscopic and ultrastructural findings. The specular microscopic image may be proposed as a typical reference image for endothelial vacuolation to eye banks.
